# Evidence-based policymaking is not like evidence-based medicine, so how far should you go to bridge the divide between evidence and policy?

**DOI:** 10.1186/s12961-017-0192-x

**Published:** 2017-04-26

**Authors:** Paul Cairney, Kathryn Oliver

**Affiliations:** 1 Politics and Public Policy at the University of Stirling, Stirling, United Kingdom; 2 Division of History and Politics, University of Stirling, Stirling, FK9 4LA United Kingdom; 3 Departmental Lecturer in Evidence-Based Social Intervention and Policy Evaluation, Oxford University, Oxford, United Kingdom; 4 Department of Social Policy and Intervention, University of Oxford, Oxford, United Kingdom

**Keywords:** Evidence-based medicine, Evidence-based policymaking, Implementation science, Improvement science, Storytelling, Policy ambiguity, Complex government, United Kingdom government, Scottish government

## Abstract

There is extensive health and public health literature on the ‘evidence-policy gap’, exploring the frustrating experiences of scientists trying to secure a response to the problems and solutions they raise and identifying the need for better evidence to reduce policymaker uncertainty. We offer a new perspective by using policy theory to propose research with greater impact, identifying the need to use persuasion to reduce ambiguity, and to adapt to multi-level policymaking systems.

We identify insights from secondary data, namely systematic reviews, critical analysis and policy theories relevant to evidence-based policymaking. The studies are drawn primarily from countries such as the United States, United Kingdom, Canada, Australia and New Zealand. We combine empirical and normative elements to identify the ways in which scientists can, do and could influence policy.

We identify two important dilemmas, for scientists and researchers, that arise from our initial advice. First, effective actors combine evidence with manipulative emotional appeals to influence the policy agenda – should scientists do the same, or would the reputational costs outweigh the policy benefits? Second, when adapting to multi-level policymaking, should scientists prioritise ‘evidence-based’ policymaking above other factors? The latter includes governance principles such the ‘co-production’ of policy between local public bodies, interest groups and service users. This process may be based primarily on values and involve actors with no commitment to a hierarchy of evidence.

We conclude that successful engagement in ‘evidence-based policymaking’ requires pragmatism, combining scientific evidence with governance principles, and persuasion to translate complex evidence into simple stories. To maximise the use of scientific evidence in health and public health policy, researchers should recognise the tendency of policymakers to base judgements on their beliefs, and shortcuts based on their emotions and familiarity with information; learn ‘where the action is’, and be prepared to engage in long-term strategies to be able to influence policy; and, in both cases, decide how far you are willing to go to persuade policymakers to act and secure a hierarchy of evidence underpinning policy. These are value-driven and political, not just ‘evidence-based’, choices.

## Background: the limits to a focus on ‘barriers’ between evidence and policy

The health sciences are well represented in studies of the ‘evidence-policy gap’, in which academics describe their attempts to overcome ‘barriers’ between the production of evidence by scientists and its use by policymakers. Oliver et al.’s [1, 2] systematic reviews draw insights from 126 health policy studies published from 2000–2012. The most frequently-reported barriers relate to problems with disseminating high quality information effectively, namely the lack of time, support, resources and incentives for scientists to engage in dissemination. These studies suggest that scientific evidence is often not presented at the correct time and scientists are unable to anticipate a demand for information to solve a very specific problem quickly. Further, policymakers lack the research skills to understand scientific evidence. More generally, there are different scientific and policymaker cultures [3]. These insights generally come from a small number of countries, including the United States, United Kingdom, Canada, Australia and New Zealand.

There are two key problems with this literature. First, very few studies draw on policy theory or other forms of knowledge of the policy process [4]. Most are based on personal practitioner perspectives generated through, for example, surveys, focus groups and academic-policymaker workshops. They generally have a particular reference point, namely evidence-based medicine (EBM) [2]. The EBM agenda is (1) to gather the best evidence on health interventions, based on a hierarchy of methods, in which randomised control trials and their systematic review are at the top, and (2) to ensure that it has a direct impact on practice, to exhort practitioners to replace bad interventions. Individual clinicians have high discretion on how to use the evidence, but based on a common ideal and understanding of evidence quality. Consequently, the studies present limited findings because (1) their source material is restricted primarily to interviewees with minimal knowledge of policymaking, and (2) potential solutions are based on a misperception of the policy process, built on EBM assumptions, not policy theory [5].

The most-frequently stated solutions to ‘barriers’ highlight the limits to such a theoretical analysis. For example, to address the problem of supply, studies highlight the need for ‘improved dissemination’ to ensure that policymakers pay attention to, and understand, the best evidence [1]. Too few studies recognise that policymakers will not share the sense that there is a hierarchy of evidence. Too many assume that better dissemination will prompt policymakers to think, like scientists, that the scientific evidence alone is persuasive or self-evident.

To address demand, the most-proposed solution is to develop scientific competence in government. Too many studies assume that it is realistic to produce a captive audience of policymakers willing to invest the time to prioritise and understand scientific evidence. This approach is at odds with the less rigid ways in which policymakers use many forms of evidence [3, 6–9].

Second, too few studies recognise the role of values in politics. Instead, an often implicit and untested assumption is that policymaking should be as ‘evidence based’ as medicine, which is at odds with the more common starting point, in the study of politics, to produce a democratic system which translates competing societal values and preferences into policy solutions. One may desire a political system based on value judgements and evidence, but should recognise and address the trade-offs between these aims, and that the production of evidence is also an inherently value-driven process.

## New directions for evidence-based policy research: two foundational questions

We address these gaps in the literature by raising two questions designed to promote discussion about pragmatic policy strategies. Each question identifies a central insight from policy studies and prompts scientists to consider how they would combine an attachment to evidence-based policymaking with other values to produce a realistic way forward.

1. *How far should scientists go to persuade policymakers to act on their evidence?*
  Policy studies suggest that actors are influential when they ‘frame’ their evidence in simple, manipulative and/or emotional terms to generate policymaker attention. Should scientists also combine evidence with emotional appeals to influence the policy agenda? Should they deny scientific uncertainty and make unequivocal claims about the implications of their evidence? Or, does this strategy produce an ethical dilemma and/or the potential to reduce long-term scientific credibility?
2. *How far should scientists go to defend a hierarchy of evidence to deliver policy solutions?*
  Initially, policy studies prompt us to identify where ‘the action’ is, to know where to engage in a complex and multi-level system in which many actors make policy in many venues. However, it does not identify the main aim of engagement. Is it to make sure that all actors prioritise ‘evidence-based’ policymaking above other factors, or to cooperate with other actors with different ideas about ‘good’ policymaking? The latter can include governance principles such as ‘localism’ and the ‘co-production’ of policy between local public bodies, interest groups and service users. This process may be based primarily on values and involve actors with no commitment to a hierarchy of evidence when they implement policy solutions.

Consequently, insights from policy studies provide practical advice and raise new dilemmas (summarised in Table 1). Our discussion takes us beyond existing debates on the hierarchy of evidence and primary role of randomised control trials (RCTs) in policy evaluation. Initially, it invites us to reflect on the extent to which a commitment to a hierarchy prompts the need for ‘fidelity’ to evidence-based solutions, limiting local policymaking discretion to ensure the correct ‘dosage’ of an intervention and maintain continuous RCT-based evaluation. Then, we should consider the role of governance principles – such as ‘localism’ and ‘co-production’ – which encourage local practitioners to modify policy solutions to meet the specific circumstances of communities and preferences articulated by service users. If we accept the value of these principles, what are the implications for ‘evidence-based’ policymaking (at least in the countries usually covered by this literature) and how might we design a process to manage the trade-offs between scientific evidence and values?

**Table 1:** New insights from policy studies raise new advice and dilemmas

	New insight from policy studies	New advice based on such insights	New dilemmas arising from such advice
How to maximise the use of evidence in policy	Too many studies focus on supplying scientific evidence to reduce uncertainty; focus instead on increasing demand for evidence by reducing ambiguity	Successful actors reduce ambiguity by, for example, framing issues in manipulative ways, using emotional language	*How far should scientists go to persuade policymakers to act on their evidence?* Should they be manipulative? This strategy may be effective, but it presents moral dilemmas and challenges a politically effective image of science as objective We identify several current responses to this dilemma
How best to understand and act effectively within the policy process	Too many studies assume that there is a policymaking ‘centre’, making policy via linear stages in a cycle; focus instead on a complex multi-level system or environment	Successful actors take the time to identify which responsibilities are delegated, ‘where the action is’ and the ‘rules of the game’ in each policymaking venue	*How far should you go to defend a hierarchy of evidence to deliver policy solutions?* Should scientists object to ‘localism’ if it undermines policies based on RCTs? Or, should they embrace the ‘co-production’ of policy with actors who reject their ‘hierarchy’ of evidential methods? We identify three main responses to this dilemma

To develop new perspectives on these questions, we identify insights from secondary data, namely systematic reviews [1], critical analysis [2] and policy theories relevant to evidence-based policymaking [3, 10, 11]. The studies are drawn primarily from countries such as the United States, United Kingdom, Canada, Australia and New Zealand. We combine empirical and normative elements to identify the ways in which scientists can, do and should try to influence policy. Our case study draws partly from an ESRC-funded study based on 30 anonymous semi-structured interviews with civil servants in the Scottish and United Kingdom governments. We use this this data to produce models of evidence-based policymaking (described in more depth in [12]), then analyse the dilemmas that they present for scientists.

To provide context for the second question, we place the possible responses that scientists could make on a spectrum, from ‘purist’, or the insistence on the primacy of RCT evidence and fidelity to successful interventions during policy implementation, to the ‘diplomatic’, treating evidence as part of a success story, encouraging the autonomy of practitioners to ‘co-produce’ and modify policy solutions to meet the values and specific circumstances of service users. We examine the implications of these practices on scientific research, outlining pragmatic responses to combine scientific evidence with governance principles.

## How to maximise the use of evidence in policy

Most policy theories explore the implications of two basic insights, namely that policymakers are limited by ‘bounded rationality’ [13] and share power with many actors in complex policymaking systems [3, 10, 14]. In part, bounded rationality relates to the fact that policymakers do not have the ability to gather and consider all evidence relevant to policy problems. Instead, they employ two shortcuts – ‘rational’, pursuing clear goals and prioritising certain sources of information, and ‘irrational’, drawing on emotions, gut feelings, beliefs and habits to make decisions quickly.

The key problem with many health studies is that they focus on the first short cut. They identify the problem of uncertainty and incomplete information, seeking to solve it by creating hierarchies of evidence and improving the supply of information to policymakers. They ignore the role of manipulation and persuasion to reduce ambiguity by establishing a dominant way to ‘frame’ policy problems. The latter can determine the demand for evidence.

From such discussions, we can provide a first key message for scientific advocates, namely to recognise the tendency of policymakers to base judgements on their well-established beliefs and shortcuts based on their emotions and familiarity with information. On that basis, consider how to reduce ambiguity, to persuade policymakers to frame a problem primarily in one particular way and, therefore, to demand scientific evidence to help solve that problem.

Persuasion or ‘framing’ strategies are effective because they appeal to the emotions and the familiar, combining facts with emotional appeals to prompt lurches of attention; telling simple and easily understood stories which manipulate people’s biases, apportioning praise and blame and highlighting the moral and political value of solutions; and recognising the importance of interpreting new scientific evidence through the lens of the beliefs and knowledge of influential actors.

Without this focus on the ways in which policymakers understand and respond to problems, scientists will be unable to exert influence, responding only to sudden policymaker demand for evidence-based solutions to a pre-defined problem [10, 15–27].

However, this first message also produces the first practical or ethical dilemma – how far should scientists go to persuade policymakers to act on their evidence? This knowledge allows us to decide how far we should go to secure influence, from the ‘pure scientist’ providing evidence with little thought for its application and the ‘honest broker’ prepared to engage with stakeholders to define policy problems [28], towards engaging in persuasion and/or manipulation to generate attention for problems and evidence-based solutions [29].

There is no definitive answer to this question. Instead, there are factors to consider, including the trade-off between (1) being manipulative and hyperbolic, to generate attention in the short term, and (2) maintaining an image of objectivity, to generate strong relationships in government based on reliability, expertise, trust and ‘independence’. Further, there are many compromise solutions, including a strategy built on framing the implications of evidence according to the stated beliefs of policymakers and building up a reputation for reliability within many policymaking venues.

### Issues with existing approaches: the focus remains on research over policy impact

In health science, many models of research impact are built on strategies which make minimal reference to policymaking, namely identifying the research question, developing a research methodology, implementing data collection, analysis and synthesis, interpreting findings, and developing research and then policy/practice recommendations. In this patrician model, the process is owned and controlled by researchers, who then advise or disseminate their work in the general direction of policymakers [30].

There is also the advocacy model, where researchers develop policy solutions and attempt to convince policymakers to adopt them [31, 32]. Most of this literature focuses on techniques (e.g. [33]) rather than risks. Gilbert [34] describes advocacy research as aiming to create social change, usually for excellent reasons, but involving tactics which can lead to researchers distorting the data (for example, giving inaccurate estimates of child abuse statistics) or presenting overly emotive cases which can ‘powerfully shape’ media coverage (for example, on rape cases). Advocacy can obscure and distort policy discussions but most discussions do not address this problem or the underlying issues of power and policy processes.

A more collaborative kind of advocacy is ‘facilitational’, in which researchers attempt to share power, responsibility and accountability for research with stakeholders. ‘Co-production’ was originally used to describe a model of public service delivery which rested on the belief that using a broad range of perspectives lead to better, fairer, more useful and more used products and services [35–38]. These beliefs have transferred to the production of knowledge through collaborative or participatory methods, which may lead to more egalitarian policymaking, through shared responsibilities [39–48].

### New collaborations between scientists and policymakers

There are many models for collaborative interactions between scientists and policymakers (e.g. [49–54]). The James Lind Alliance [55] has long been active in the ‘co-production’ of research agendas between patients, clinicians and researchers; the National Institute for Health and Clinical Excellence (NICE) has used stakeholder groups to generate clinical and research recommendations and priorities for nearly two decades [56]. Collaborative research practices tend to fall into two groups – using each other’s skills and expertise for relatively discrete sections of the research process (collaborative) and engaging in a whole process of equal control and decision-making (co-productive) – although these labels are not consistently applied. Both describe a ceding of control by researchers, over some or all of the research process, to stakeholders, including policymakers, to a greater or lesser degree [57, 58].

Little research has been performed on the strengths, risks and implications for research, researchers and policy of these models of interactions [34, 43]. Proponents tend to promote their models as more democratic or as producing higher quality research, although generally this is an ideological rather than empirically-grounded stance. Proponents of the patrician or neutral models are presumably wary about stating their position, as co-productive research is increasingly claimed as the vehicle for research ‘impact’. Some commentators identify risks to researchers in lessening control or increasing advocacy [59, 60], but this has not translated into a considered discussion of the implications for the evidence-based policy movement. Few studies consider the insights we highlight about the roles of policy actors, the consequent judgments about what counts as evidence and why, and how decisions are made.

What most of these models have in common is that they are modifications of the research process, not responses to the policy process. The disinclination of many scientists to engage with the policy world, even amongst the most collaboratively minded, is revealing. It highlights an unresolved issue about the extent to which a systematic research-driven model can be adapted and improved to reflect the need to compete with advocates of other forms of evidence to secure the attention and support of policymakers.

In summary, scientists have developed different answers to the question ‘how far should you go?’ These answers correspond to ways in which scientists and policy actors interact, from disseminator/receiver to equal co-producers. However, these interactions have not yet been examined through the lens of policy theory, nor have empirical comparative descriptions enabled us to determine what types of interactions work best, for which purposes and under which circumstances.

## How best to understand and act effectively within the policy process

Any competition to frame issues takes place in a complex policymaking environment or system [61]. This context is not described well by the ‘policy cycle’ – an understanding built on the idea that policymaking can be separated into discrete stages (agenda setting, policy formulation, implementation, legitimation, evaluation and succession) – which remains popular within health scholarship despite being rejected by policy scholars as a poor explanation of policymaking [2, 3, 10, 14, 62–66].

In its place are theories which identify a policy ‘environment’ which relates to the interaction between five factors, namely the many policy actors operating in multi-level systems, pursuing ideas (using their beliefs and knowledge to influence how policymakers think), adapting to institutions (the formal and informal rules of political systems and organisations), networks (between policymakers and influential actors), and the impact of socioeconomic context and events [3, 27, 67–71]. Further, some theories identify complex policymaking systems in which, for example, the same inputs of evidence can receive no, or disproportionate attention, and policy outcomes often ‘emerge’ in the absence of central government control, which makes it difficult to know how, and to whom, to present evidence [3, 72].

This analytical shift from cycles to environments and systems helps us reject the notion that there is a core group of policymakers at the heart of the process, analysing and making policy from the ‘top down’, and that scientists promoting evidence can focus their efforts at a single point of problem definition or policy formulation [3].

Rather, scientists compete with many influential actors to present evidence to secure a policymaker audience at many levels of government. Support for evidence-based solutions varies according to which organisation takes the lead and how its rules encourage policymakers to understand the problem. Some networks are close-knit and difficult to access because bureaucracies have operating procedures that favour some sources of evidence and participants over others. There is a language in institutions and networks that takes time to learn. It indicates which ways of thinking are in good ‘currency’, including a dominant way to ‘frame’ a problem and limit discussion to certain solutions. Well-established beliefs provide the context for policymaking – new evidence on the effectiveness of a policy solution has to be accompanied by a shift of attention and successful persuasion. Social or economic ‘crises’ can prompt lurches of attention from one issue to another, and some evidence can be used to encourage that shift. However, most policy change is minor; it is rare for events to combine with new evidence to produce quick and radical policy change [10].

From such discussions, we can provide a second key message for scientific advocates – learn ‘where the action is’, and be prepared to engage in a long-term strategy to be in a position to influence policy. In other words, identify ‘the action’ at several levels of government, learn the ‘rules of the game’ in institutions and networks (or the ‘venues’ in which policymaking takes place), form coalitions with like-minded actors, and work with the ‘policy entrepreneurs’ possessing the skills to exploit ‘windows of opportunity’ to give policymakers the motive and opportunity to adopt new solutions [3, 8, 23, 24, 27, 73–78].

In most cases, these coalitions will be with many actors with different ways of thinking about policymaking, engaging in politics to turn their beliefs and values into policy, often unaware or unsupportive of EBM’s hierarchy of evidence, and likelier to refer to governance principles other than evidence-based policymaking.

Consequently, this second message produces a second practical and ethical dilemma – how far should you go to defend a hierarchy of evidence to deliver policy solutions? It involves the extent to which policy actors should accept and adapt to policymaking complexity and the diffusion of power, or seek to change some aspects and assert their claim for greater power.

For example, central governments have some incentives to try to reassert central control because they are held accountable in elections and parliaments for policy outcomes, regardless of their responsibility [14]. Yet, they also recognise the need to share decision-making with actors at multiple levels of government, including at the point of policy delivery. This produces a form of government in which the co-production of policy solutions is based partly on evidence and partly on the principles underpinning local governance, including ‘localism’ (central governments recognise the role of partly autonomous local authorities or partnerships) and service user-driven services (based on their values and preferences) [12].

In that context of multi-level and delegated policymaking, scientists may support phrases such as ‘co-production of research’ superficially (who would not want to involve all relevant actors in the production of knowledge?), but also recognise the need for tough choices to ensure the application of evidence-based policy solutions. They need to engage with two debates simultaneously, on EBM and localism.

First, not all actors adhere to the EBM ideal [79–83]. There are several different reasons to expect a different approach, as described below.

- Policymakers may not care that evidential hierarchies exist, preferring a variety of sources of knowledge [3, 6, 84]. While RCTs enjoy high status in some units in government and key champions [85], in others they may be used primarily to reinforce existing agendas [8]. Indeed, policymakers can appropriate research findings and reduce academic criticism of evidence use by commissioning evidence directly and/or seconding academics to government roles [86]. Or, policymakers may accept scientific evidence on the size of a problem and recognise the potential value of solutions from the same academic source, without finding a politically feasible way to introduce them (see, for example, [87] on the limited receptivity to measures to reduce health inequalities).
- Some advocates of RCTs recommend greater reflection on scientific values in politics rather than the assumption that hierarchies of evidence are necessarily valid [88, 89].
- EBM contrasts with an adherence to practice-based evidence, which includes the experiential knowledge of practitioners, feedback from service users and a greater focus on supporting different voices ([41, 44]).
- Some scholars argue that complex policymaking systems defy control and, therefore, are not conducive to the use of RCTs (see [61]). Instead, we use other methods, such as process tracing [90], large-scale quantitative evaluation designs like difference in differences, or case studies, to highlight the interaction between a large number of factors which combine to produce an outcome, the result of which cannot be linked simply to the independent effects of each factor.

Second, central governments face the trade-off between national and local policymaking [91]. Their national strategies can include reference to uniform delivery standards, to avoid a ‘postcode lottery’, and encourage local autonomy and policy flexibility, to recognise the legitimacy of local policymaking and encourage community or service user participation. For some interventions, they may favour uniformity of delivery and fidelity to a programme. For others, they express a preference for localism built on arguments, including one size does not fit all, local authorities have their own electoral mandates, a policy will not succeed without local ‘ownership’, and/or local public bodies need to adapt to quickly changing local circumstances.

Consequently, there is an inextricable link between debates on the selection of evidence-based solutions/interventions and the correct choice of governance arrangements. This involves a two-part decision in which policymakers combine (1) their chosen method to identify and gather knowledge of successful policy solutions, with (2) their judgement on how to work with other actors to ‘scale up’ success.

In this context, consider the scale of the task to ensure the primacy of RCTs and/or a hierarchy of evidence in policymaking. It involves (1) persuading many actors in many central and local venues that (2) there is a well-established best way to produce scientific evidence, and therefore (3) that scientific forms of knowledge should be prioritised in policymaking. This is a two-pronged dilemma – should you devote necessarily high resources to this line of persuasion, and should you go to great lengths to dismiss competing understandings of good policymaking?

## Three responses to this dilemma

To demonstrate the issues central to dilemma 2, we outline a spectrum of approaches to answer the ‘how do you scale up success?’ question (see [12] for a summary table). It allows us to demonstrate the implications for evidential hierarchies when power is spread across multiple levels of government. We show the constraining effect of a rigid attachment to ‘fidelity’ to policy interventions on the ability of local communities to modify policy solutions to reflect their values and preferences. This outcome may only be appropriate in some cases. We compare it with an approach that favours local flexibility and governance principles at the expense of scientific evidence. The contrast allows us to highlight the political choices with which advocates of evidential hierarchies need to engage. We use examples of initiatives pursued by the United Kingdom and Scottish central governments, each of which enact or negotiate the delivery of policy interventions with a variety of elected local authorities and unelected health and social care organisations.

### Approach 1: prioritise RCTs and focus on fidelity and dosage

The primary aim of movements such as ‘implementation science’ is to gather the best evidence on health interventions and ensure that it has a direct impact on practice [1, 92]. One should not exaggerate the top-down nature of EBM [93, 94]. However, we can use its key tenets to produce one ideal-type approach:

1. Gather evidence of effectiveness with reference to a hierarchy of evidence and evidence gathering, with systematic reviews and RCTs at the top.
2. ‘Scale-up’ best practice by introducing the same model in each area, require ‘fidelity’ to administer the correct dosage and allow us to measure its effectiveness with RCTs.
3. Prioritise the administration of the correct dosage of the ‘active ingredient’.

This language of providing the correct dosage is common in medicine, based on the use of evidence to measure the effectiveness of the active ingredient (e.g. in ibuprofen it is isobutylphenyl), with less focus on the delivery system (e.g. the gelatine capsule). However, in health and social care it refers to the interaction between practitioners and service users – the ‘dosage’ of an intervention by a practitioner is harder to distinguish from the ‘delivery system’.

The classic example is the Family Nurse Partnership (FNP). It began in the United States as the Nurse–Family Partnership, designed to engage nurses with first time mothers (deemed to be at relatively high risk of poor life chances) once per month from pregnancy until the child is two. The United States’ Coalition for Evidence-Based Policy [95] gave it ‘top tier’ status, which describes “*Interventions shown in well-designed and implemented randomised controlled trials, preferably conducted in typical community settings, to produce sizable, sustained benefits to participants and/or society*”. Trials have been conducted since 1977, producing 18 peer-reviewed articles in elite journals (such as *Journal of the American Medical Association*), with at least two non-United States studies [96]. It was rolled out in England to 9000 mothers, with reference to its high cost effectiveness and ‘strong evidence base’, which would be enhanced by an RCT to evaluate its effect in a new country ([97]; the first evaluation was unfavourable - [98]. The FNP requires fidelity to the United States programme – it can only be used if users agree to the licensing conditions – based on evaluation results which showed that the programme was most effective when provided by nurses/midwives and using a license “*setting out core model elements covering clinical delivery, staff competencies and organisational standards to ensure it is delivered well*” ([99], p. 6). Fidelity is a requirement because, “*If evidence-based programmes are diluted or compromised when implemented, research shows that they are unlikely to replicate the benefits*” ([99], p. 6) and the FNP website outlines ‘fidelity goals’ which resemble those for dosages of medicine.

### Approach 2: storytelling

A storytelling approach suggests that you:

1. Gather evidence of effectiveness with reference to practitioner and service user testimony.
2. ‘Scale-up’ best practice by telling stories based on your experience and inviting other people to learn from them.
3. Prioritise key principles such as respect for service user experiences and a conducive environment in which to deliver public services respectfully.

My Home Life is a key example. It began as an English initiative, also developed in Scotland, “*to promote quality of life for those living, dying, visiting and working in care homes for older people through relationship-centred and evidence-based practice*” (http://myhomelife.uws.ac.uk/scotland/). With this approach, much derives from individual feedback, with a focus on the richness of experience. The result is a set of principles to inform future practice, not a specific intervention with a correct dosage. The principles are deliberately broad to allow practitioners and service users to make sense of them in specific settings [100, 101]. This approach contrasts with the FNP’s requirement to follow the model closely and gather quantitative data to measure fidelity. With My Home Life, there is no model and practitioners and service users draw on their experiences to guide future practice and develop favourable institutional cultures.

### Approach 3: improvement science as a pragmatic compromise?

The comparison prompts us to recognise that there are no easy compromises between approaches to policy delivery that have fundamental differences. For example, the distinction between dosage and delivery system allows us to focus on the dilemmas in multi-level policymaking that cannot be solved with reference to evidence alone, because they involve prioritising intervention effectiveness versus governance principles based on practitioner and service user experience and values. There is a clear trade-off in practice between (a) a rigid attachment to EBM during policy delivery, and (b) the governance principles that many governments favour when promoting a storytelling approach [12].

In that context, we should ask how far health scientists should go to ensure the correct delivery of an intervention. Should they be uncompromising, or seek ways to promote evidence-based policy and practices such as co-production in evidence gathering and delivery? At one end of the spectrum, in focusing on the evidence hierarchy, researchers assert their authority to describe reality [57], claiming that empirical evidence derived and produced by them is the best account of how the world may be manipulated.

In reality, few such researchers exist. Co-produced RCTs, amongst other designs, are becoming the norm [102, 103]. Yet, such developments do not resolve the problems we raise, because there is a big difference between co-production based on RCTs and the development of ‘improvement science’ approaches. The latter are often built on a narrative of RCT scepticism and faith in the autonomy of (professionally trained) local practitioners [12]. They have the advantage of pragmatism, and have generated significant support among the United Kingdom and Scottish governments, but note how far they take us from approach 1 when they suggest that you:

1. Gather evidence of effectiveness based on a mix of evidence, from RCTs to experiential data.
2. ‘Scale-up’ best practice through local experimentation and learning.
3. Prioritise the use of a specific improvement method, training practitioners before they experiment and decide how best to turn evidence into practice.

The Early Years Collaborative (EYC) (Scotland) is a key example. ‘Collaborative’ refers to a group of organisations engaging on a problem, drawing on the ‘sound science’ on how to reduce costs or improve outcomes, which exists but “*lies fallow and unused in daily work*” ([104], p. 1). Participants identify an aim, measures of success and the changes to test, then gather quantitative data on their effects, using a form of continuous learning summed up by a ‘Plan-Do-Study-Act’ cycle [104].

The EYC is an attempt, from 2012, to use the Institute for Healthcare Improvement’s (IHI) method for single organisations to coordinate a multi-agency project, working with local and health authorities through ‘community planning partnerships’. The general assumption is that, in some areas, there is no set of known, effective interventions. Rather, an important strand of this approach is learning as you go, with a long-term aim to gather comparable data on local practices to aid learning, supplemented by ‘word of mouth’ measures of success and ad hoc decisions to expand projects they feel are successful. This is justified with reference to the poor alternatives, such as the excessive gaps between the RCT evidence on a problem and resultant practice [105]. The stated ‘theory of change’ is that, if you engage and train the workforce in the IHI method, they will use it to address key policy challenges [106]. Further, rather than attempting to direct local activities, a small central government team helps practitioners use a ‘toolkit’ for improvement. When they discuss ‘scaling up’ practices to the national level, this refers more to the IHI delivery method than the dosage of specific interventions.

Other improvement science-inspired approaches have become very common in health sciences, especially amongst projects such as the Collaborations for Leadership in Health Research and Care (CLAHRC) (see, e.g. [53, 107–109]). These approaches all have a common belief in progressive improvement through mutual learning, but note that, in many examples, the research agenda is set by clinical or research leads, aiming to address identified local issues and to produce recommendations [107].

In other words, the focus of improvement science scholarship tends towards discussion of how to optimise delivery of evidence-based practice, and tends not to discuss the importance of the local. The EYC reverses this emphasis, using scholarship as one of many sources of information and focusing primarily on the ‘assets’ of practitioners and service users [12]. Consequently, initiatives such as improvement science appear to offer pragmatic solutions to the gap between approaches 1 and 2, but only because they mean very different things to different people. The adoption of improvement science is only one step towards negotiating the trade-offs between RCT-driven and story-telling approaches.

## Conclusion

We identify two implications of policy theory for scientists, namely to address ambiguity and complexity. From these, we identify key messages for policy actors. First, be realistic about how policy decisions are made, and adapt persuasion techniques accordingly. This requires scientists to decide how far they should go to persuade policymakers: when does persuasion tip into cynical manipulation? Second, be prepared to engage in long-term relationships to build coalitions for change. This prompts scientists to decide when to defend the ‘hierarchy’ or primacy of evidence and when to accept the value of other sources of ‘good’ policymaking. Further, while improvement science appears to offer a happy marriage between these approaches, in practice it offers only one step towards negotiating the trade-offs between RCT-led and story-telling approaches.

Our discussion takes scientists away from the lazy assumption that elected policymakers are the villains of this piece because they, for example, do not understand RCTs and the need for RCT-driven evaluations, do not recognise a hierarchy of evidence in which the systematic review of RCTs represents the gold standard, and/or are unwilling to overcome ethical dilemmas about who gets to be in/out of the control group of a promising intervention. There are also academics and professions who remain sceptical of the value of RCTs in policymaking, have different views on the hierarchy of evidence and/or who promote different ways to gather and use comparable policy-relevant evidence [83].

Evidence-based policymaking is not just about the need for policymakers to understand how evidence is produced and should be used. It is also about the need for academics to reflect on the assumptions they make about the best ways to gather evidence and put the results into practice, in a political environment where other people may not share, or even know about, their understanding of the world; and the difference between the identification of evidence on the success of an intervention, in one place and one point in time (or several such instances), and the political choice to roll it out, based on the assumption that national governments are best placed to spread that success throughout the country.

We have largely discussed extremes or ideal types to highlight key issues. In practice, people may be willing to compromise or produce pragmatic responses to the need to adapt research methods to real world situations. In that context, this kind of discussion should help clarify why scientists may need to work with policymakers or practitioners to produce a solution that each actor can support and why that solution may not be ‘evidence based’ in the way that scientists understand that phrase.
